# Carbolic Acid for the Cure of Internal Piles

**Published:** 1879-08

**Authors:** 


### CARBOLIC ACID INJECTIONS FOR THE CURE OF INTERNAL PILES.

In a discussion in the surgical section of the American Medical Association (May 7), Drs. Z. R. Weist, J. W. Murphy, W. H. Brown, and A. B. Cook testified to the safety and efficiency of this method for the cure of internal piles. The acid is injected into the substance of the tumor, either pure or diluted with glycerine or alcohol. It will be remembered that Dr. Andrews, of Chicago, is endeavoring to collect statistics on this subject, in order to determine the safety of the acid treatment compared with the older methods, viz., the ligature, the clamp, and the cautery. Until recently the carbolic acid treatment was resorted to by advertising quacks, who sold it as a secret remedy.—*The Medical Herald, Louisville.*

